# Typing and modeling of hepatocellular carcinoma based on disulfidptosis-related amino acid metabolism genes for predicting prognosis and guiding individualized treatment

**DOI:** 10.3389/fonc.2023.1204335

**Published:** 2023-08-11

**Authors:** Xuenuo Chen, Zhijian Wang, Yilin Wu, Yinghua Lan, Yongguo Li

**Affiliations:** ^1^ Department of Infectious Diseases, The First Affiliated Hospital of Chongqing Medical University, Chongqing, China; ^2^ Department of Gastroenterology, The First Affiliated Hospital of Chongqing Medical University, Chongqing, China; ^3^ Department of Infectious Diseases, The Second Affiliated Hospital of Chongqing Medical University, Chongqing, China

**Keywords:** hepatocellular carcinoma (HCC), disulfidptosis, amino acid metabolism, immune microenvironment, microsatellite instability, prognostic signature

## Abstract

**Introduction:**

Hepatocellular carcinoma (HCC) is the most common type of cancer worldwide and is a major public health problem in the 21st century. Disulfidopathy, a novel cystine-associated programmed cell death, plays complex roles in various tumors. However, the relationship between disulfidoptosis and prognosis in patients with HCC remains unclear. This study aimed to explore the relationship between disulfideptosis and the prognosis of liver cancer and to develop a prognostic model based on amino acid metabolism and disulfideptosis genes.

**Methods:**

We downloaded the clinicopathological information and gene expression data of patients with HCC from The Cancer Genome Atlas (TCGA) and Gene Expression Omnibus (GEO) databases and classified them into different molecular subtypes based on the expression patterns of disulfidoptosis-associated amino acid metabolism genes (DRAGs). Patients were then classified into different gene subtypes using the differential genes between the molecular subtypes, and the predictive value of staging was assessed using survival and clinicopathological analyses. Subsequently, risk prognosis models were constructed based on Cox regression analysis to assess patient prognosis, receiver operating characteristic (ROC) curves, somatic mutations, microsatellite instability, tumor microenvironment, and sensitivity to antitumor therapeutic agents.

**Results:**

Patients were classified into two subtypes based on differential DRAGs gene expression, with cluster B having a better survival outcome than cluster A. Three gene subtypes were identified based on the differential genes between the two DRAGs molecular subtypes. The patients in cluster B had the best prognosis, whereas those in cluster C had the worst prognosis. The heat map showed better consistency in the patient subtypes obtained using both typing methods. We screened six valuable genes and constructed a prognostic signature. By scoring, we found that patients in the low-risk group had a better prognosis, higher immune scores, and more abundant immune-related pathways compared to the high-risk group, which was consistent with the tumor subtype results.

**Discussion:**

In conclusion, we developed a prognostic signature of disulfidptosis-related amino acid metabolism genes to assist clinicians in predicting the survival of patients with HCC and provide a reference value for targeted therapy and immunotherapy for HCC.

## 1 Introduction

According to the International Report on Oncology Statistics, primary liver cancer is the fourth most lethal cancer (1). Hepatocellular carcinoma (HCC) accounts for approximately 80% of primary liver cancers and is the most common type (2) Currently, The main treatment options include local radiofrequency ablation, surgery, radiotherapy, and immunotherapy, which have improved patient survival (3). However, a high degree of heterogeneity among patients has led to different outcomes in most patients with HCC after comprehensive treatment. The complexity and polygenicity of tumors may explain the limitations of current treatment options; therefore, there is an urgent need to identify valuable prognostic models to evaluate targeted and more effective treatments for individual patients with liver cancer. Recent studies have shown that the intracellular accumulation of disulfides induces a stress response that leads to disulfidosis, a new form of programmed cell death (4). Normally, cancer cells rely on the amino acid transporter protein SLC7A11 to transport cystine intracellularly to promote tumor growth; however, cystine is a disulfide that may have cytotoxic effects. To regulate this balance, cells rapidly convert toxic disulfides into nontoxic molecules using NADPH (4, 5). NADPH is mainly produced by glucose metabolism; therefore, when tumor cells lack glucose, it can trigger disulfidaptosis in tumor cells, which in turn inhibits tumor growth. However, this process is not cytotoxic to normal tissues (6). Since the concept of disulfidoptosis was first proposed, it has attracted considerable attention from the medical community, particularly in the field of tumor therapy. Therefore, understanding the state of disulfidoptosis in different HCC populations is important for exploring targeted therapies for HCC.

Metabolic reprogramming was found to be the main feature in the analysis of three common tumor metabolites, and amino acid metabolism plays an important role in tumor development (7). Several studies have shown that genes related to amino acid metabolism act as metabolic regulators that meet the demands of rapid tumor cell proliferation. For example, glutaminase 2 (GLS2) regulates glutamine metabolism by binding to P53, decreasing the antioxidant capacity of reduced glutathione and reducing ROS levels, which in turn enhances the tumor-suppressive function of P53 (8, 9). The oncogenic transcription factor cMyc in tumor cells acts on serine hydroxymethyltransferase (SHMT) to promote tumor proliferation by depleting serine (10, 11). Argininosuccinate synthase (ASS), a key enzyme in the arginine synthesis pathway, is defective in various tumors, including HCC. Some investigators have developed arginine degradation therapies based on this feature, which are used to treat patients with this group of tumors (12). Exploration of amino acid metabolism is important for the prevention and treatment of HCC. In addition, the immune microenvironment of HCC provides a suitable growth environment for tumor development (13), and chimeric antigen receptor (CAR) T-cell therapies and monoclonal antibodies against programmed cell death protein 1 (PD-1), thus improving the prognosis of patients with HCC by altering the pathways where immune cell checkpoints are located and thus improve the prognosis of patients with HCC (14). However, it remains unclear whether amino acid metabolism-related genes are involved in immune regulation in HCC. Therefore, further studies are required to determine the prognosis of patients with HCC.

**Figure 1:**
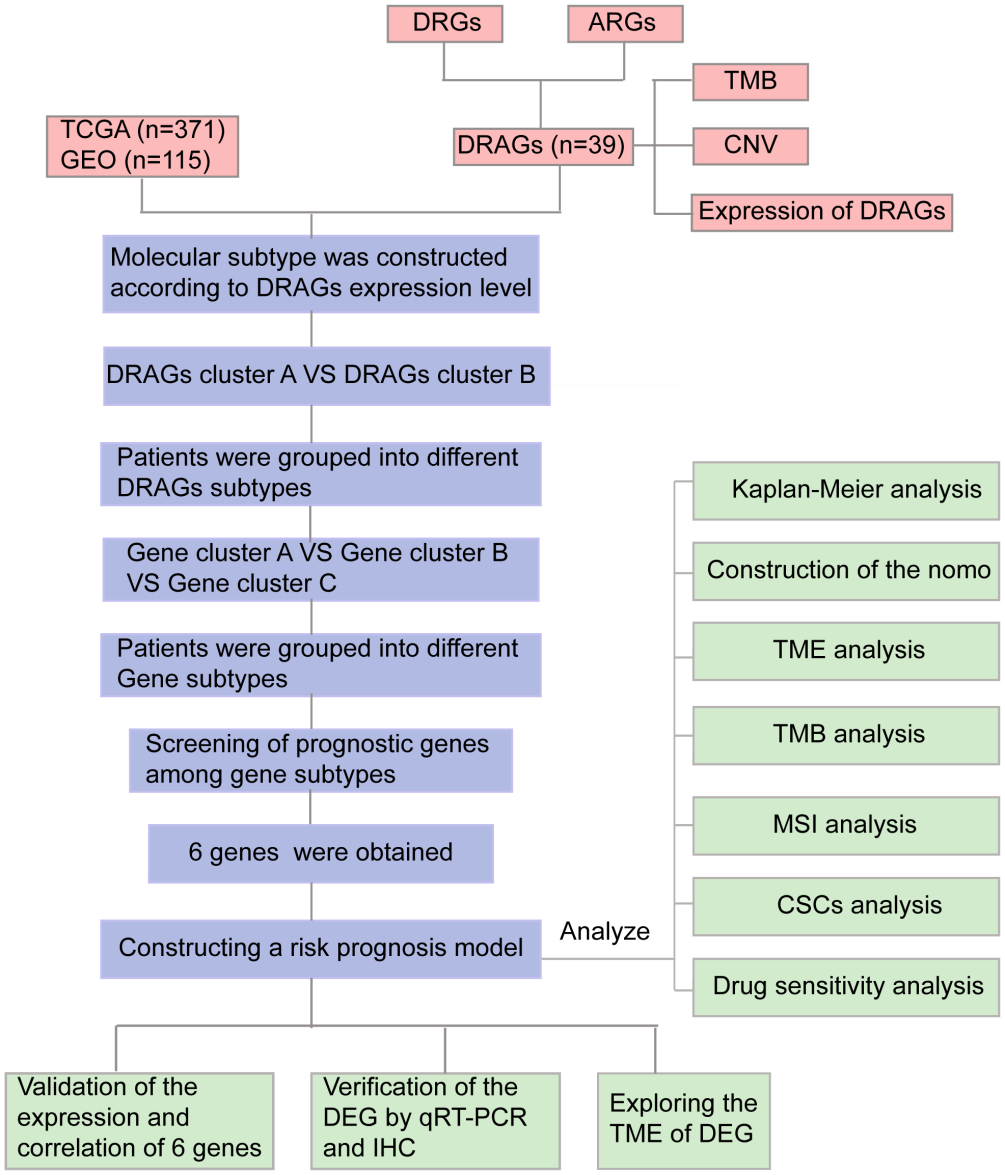
Flow chart of our study. DRGs, disulfidptosis related genes; ARGs, amino acid metabolism-related genes; DRAGs, disulfidptosis related glycolytic genes TCGA, The Cancer Genome Atlas; GEO, Gene Expression Omnibus; TMB, tumor mutational burden; CNV, copy number variation; GSEA, Gene Set Enrichment Analyses; TME, tumor microenvironment; MSI, microsatellite instability; CSCs, cancer stem cells; IHC, immunohistochemical; DEGs, Differentially expressed genes.

The relationship between disulfidoptosis and amino acid metabolism, two important processes in tumor development, has received increasing attention. Amino acid metabolic reprogramming is an essential feature of abnormal metabolic changes in tumors that can endow cancer cells with the ability to proliferate rapidly. However, as a new form of programmed death, disulfidoptosis is closely related to cysteine/glutamine acid metabolism. In this study, we developed a prognostic signature combining disulfide-related genes (DRGs) and amino acid metabolism-related genes (ARGs), which is valuable for assessing the prognosis of patients with HCC. A flowchart of the study is presented in **Figure 1**.

## 2 Materials and methods

### 2.1 Collation and collection of data

First, we downloaded clinicopathological information, gene expression matrix data, and somatic mutation data of patients with HCC from TCGA database. Another set of data containing the survival information of patients with HCC was downloaded from the GEO database, and joint analysis of data from multiple databases helped reduce the heterogeneity of individual datasets. The GSE76427 and TCGA-LIHC data downloaded from the GEO database were combined using the “merge” package, and the “sva” package in R language was used to correct for differences and normalize for different sequencing batches, excluding patients with missing survival information. We finally obtained 371 patients with HCC from TCGA database and 115 patients from the GEO database, which were used for subsequent analysis.

### 2.2 Clinical sample collection

We randomly collected 23 pairs of fresh HCC and adjacent normal tissue samples were randomly collected from the First Hospital of Chongqing Medical University (Chongqing, China) between February and March 2023. Twenty pairs of paraffin-embedded sections of HCC and paracancerous tissues were retrospectively collected from patients with HCC who underwent surgery at the First Affiliated Hospital of Chongqing Medical University between June 2022 and December 2022 at the Diagnostic Pathology Center of Chongqing Medical University (Chongqing, China). None of the patients in our study underwent radiotherapy, chemotherapy, or immunotherapy before surgery. This study was approved by the Ethics Committee of the First Affiliated Hospital of Chongqing Medical University and all patients signed an informed consent form before the study.

#### 2.2.1 Quantitative real time PCR (qRT-PCR)

Total RNA was extracted from 23 pairs of fresh HCC and paraneoplastic tissues using the TRIZOL reagent (Takara Biotechnology Co., Ltd., Dalian, China) according to the manufacturer’s instructions. total RNA was reverse transcribed into cDNA using the PrimeScrip™ RT kit (Takara Biotechnology Co., Ltd.). The RNA was reverse-transcribed into cDNA. Polymerase chain reaction (PCR) was performed to detect the mRNA expression of CD8A in HCC and paraneoplastic tissues according to the manufacturer’s instructions. The amplification product was designed by Takara Biotechnology Co., Ltd. with the following sequence: CD8A: forward,5′-TCATGGCCTTACCAGTGACC-3, ‘ and reverse, 5′-AGGTTCCAGGTCCGATCC-3′; β-actin: forward, 5′-AGAAAATCTGGC ACCACACCT-3,’ and reverse, 5′-GATAGCACA GCCTGGATAGCA-3. ‘ Expression was normalized to that of β-actin and relative expression was calculated using the 2-ΔΔCt method (15).

### 2.3 Immunohistochemical staining (IHC)

Immunohistochemical staining was performed on 20 paraffin-embedded HCC and normal paracancerous tissue samples. The specific experiments were performed as previously described (16). Anti-human CD8a antibody (1:5000, Proteintech) was used to incubate the tissues overnight at 4°C. After application of the appropriate secondary antibody, the labeled antigen was visualized using a standard 3,3’-diaminobenzidine (DAB) protocol. The slides were stained with hematoxylin. Two pathologists evaluated the staining results in a double-blind manner. The specimens were divided into high- and low-expression groups according to the degree of staining, and the number of cells in each group was counted.

### 2.4 Differential analysis of DRAGs, genomic features

The expression differences and prognostic characteristics of DRAGs in HCC samples and normal tissues were assessed by the “limma” and “survival” packages of R language. Then, the frequency and type of mutations of 39 DRAGs in patients with HCC were analyzed by the “maftools” package, and the results were presented as “waterfall plots.” In addition, the somatic copy number variation (CNV) frequencies of the above genes were shown by “bubble plots,” and the sites where the mutations occurred were shown by “circle plots”.

### 2.5 Consensus clustering analysis of DRAGs

We retrieved 14 disulfidoptosis-associated genes (**Table S1**) from the relevant literature on the MSigDB website (https://www.gsea-msigdb.org/gsea/msigdb/), and 374 ARGs (**Table S2**) were extracted from the MSigDB website (https://www.gsea-msigdb.org/gsea/msigdb/). Then, we normalized the data from TCGA and GEO databases using the “limma” package of R language, and obtained 39 disulfidoptosis-related glycolytic genes (DRAGs) with the screening condition of |cor| > 0.55. Consensus clustering analysis was performed using the ConsensusClusterPlus R language package to classify the enrolled HCC patients into different molecular subtypes according to differences in gene expression. Intragroup associations were enhanced, and intergroup associations were reduced after clustering. Heterogeneity between the two groups was assessed using principal component analysis (PCA). To assess the value of consistent cluster analysis in the management of patients with HCC, we compared between-group differences in clinicopathological characteristics of patients with different subtypes by heat map, which was drawn using the R language package “survival” and “survminer.” Kaplan-Meier (K-M) curves were used to determine survival differences between the two subtypes. The Kyoto Encyclopedia of Genes and Genomes (KEGG) was used for functional variation analysis (GSVA). In addition, differences in immune cell infiltration were analyzed using single-sample gene set enrichment analysis (ssGSEA) to understand the differences in immune microenvironments between the groups.

### 2.6 Screening, functional analysis and prognostic analysis of differential genes between molecular subtypes of DRAGs

Differentially expressed genes (DEGs) among the molecular subtypes of DRAGs were screened using the “limma” package in R language with FDR<0.05 and |log2fold change (FC)|≥0.585 as criteria. Functional enrichment analysis was performed using GO and KEGG to further explore the potential gene functions and enrichment pathways of DRAGs, functional enrichment analysis was performed using Gene Ontology (GO) and KEGG.

Next, differential genes with prognostic value between the two subtypes were screened using univariate Cox regression analysis, and the patients were classified into different gene subtypes based on these genes. Survival analysis was performed using K-M to verify the prognostic differences between different gene subtypes. In addition, differences in the clinicopathological characteristics between patients with different gene subtypes were evaluated to guide the development of targeted therapies.

### 2.7 Construction of prognostic signature

First, genes with prognostic value were screened using univariate Cox regression analysis for the above differential genes, and the accuracy of the signature was improved using LASSO regression analysis. Independent prognostic factors associated with HCC were screened based on multivariate Cox regression analysis, and the risk score was calculated using the multivariate Cox regression coefficients and the expression of DRAGs in patients with HCC. Then, the prognostic signature was constructed. The scoring formula was as follows:


risk score=∑(Expi ∗ coefi)

where Expi and coefi represent gene expression and regression coefficients, respectively. Subsequently, all patients with HCC were randomly divided into training and test groups in a 1:1 ratio. Patients were further divided into high- and low-risk groups based on their median prognostic scores.

### 2.8 Analysis and validation of clinical relevance of prognostic signature

First, we calculated the differences in risk scores across DRAGs molecular subtypes and gene clusters to assess whether the risk score retained its predictive power across subgroups. Differential expression maps of DRAGs molecules between the high- and low-risk groups were constructed using the ggplot2 package. The prognostic value of clinicopathological factors and risk scores was assessed using Cox regression analysis. Next, survival differences between patients in the high- and low-risk groups were assessed using the K-M survival analysis, and ROC curves were plotted to assess the diagnostic value of this scoring system. The accuracy of the results was validated in a test group.

### 2.9 Creation and verification of nomogram

In order to evaluate the prognostic characteristics of patients at 1-, 3- and 5-year, the “rms” package and the “regplot” package of R language were used to construct the nomogram by combining clinical information such as gender, age, tumor stage and risk scores of patients. Each patient’s clinical information corresponded to a score and the total score was the sum of each index used for the scoring system of the nomogram. Finally, the scores were used to assess the probability of survival at 1-,3-,5-year intervals.

### 2.10 Exploration of tumor immune microenvironment

The main characteristics of the tumor immune microenvironment include the level of immune cell infiltration, expression profile of immune checkpoints, and activity of anti-cancer immune responses. First, we assessed the correlation between risk scores and the proportion of immune cell infiltration in patients with HCC using Spearman’s correlation analysis. We also used the “CIBERSORT” package in R to quantify the enrichment of different immune cells in each tumor sample and analyzed the relationship between genes and immune cells in the signature. To further understand the differences in the tumor microenvironment (TME) between the high- and low-risk groups and their relevance to immunotherapy, we evaluated the differences in immune checkpoint expression between the high- and low-risk groups. In addition, the ESTIMATE algorithm was applied to calculate the stromal, immune, and estimated scores in the two tumor groups, reflecting the degree of stromal and immune cell infiltration and tumor purity for each risk group, respectively, and a violin plot was used to visualize the differences in TME scores between the high- and low-risk groups. In addition, we assessed the enrichment of immune-related pathways in the high- and low-risk groups using GSEA and the activity of the seven steps of the anticancer immune response using ssGSEA to understand the role of risk scores in the tumor immune microenvironment and thus assess tumor prognosis (17, 18).

### 2.11 Genomic characterization and drug sensitivity analysis in prognostic signature

We applied the mutation data downloaded from TCGA to HCC and analyzed the tumor mutation burden (TMB) and major mutation types in the different risk groups. TMB has emerged as a biomarker to predict the efficacy of immunotherapy (19). In addition, it has been shown that microsatellite instability (MSI) is associated with tumorigenesis, generally caused by DNA replication defects (20). We used MSI analysis between different risk groups as a reference for prognostic assessment. The poor prognosis of HCC and the emergence of drug resistance are closely related, and studies on tumor stem cells (CSCs) indicate that tumor development is driven by a fraction of stem cells; therefore, it is crucial to explore the stemness of HCC stem cells (21). We assessed the degree of similarity between tumors and stem cells by calculating mRNAsi to quantify the relationship between CSCs and risk scores. Next, to clarify the efficacy of chemotherapeutic drugs in patients with risk subgroups of HCC, we calculated the drug concentration (IC50) values when half of the cells were induced to undergo apoptosis by drugs used for HCC treatment using the “pRRophetic” package.

### 2.12 Statistical analysis

We analyzed the data using the R language software (version 4.2.2), performed *t*-tests for normally distributed data, and applied Spearman’s test for correlation analysis. GraphPad Prism software (version 8.0.1) was used for plotting the images, with P<0.05 as the threshold of significance for all statistical analyses.

## 3 Results

### 3.1 Characterization and expression of DRAGs mutations in HCC

**Figure 2:**
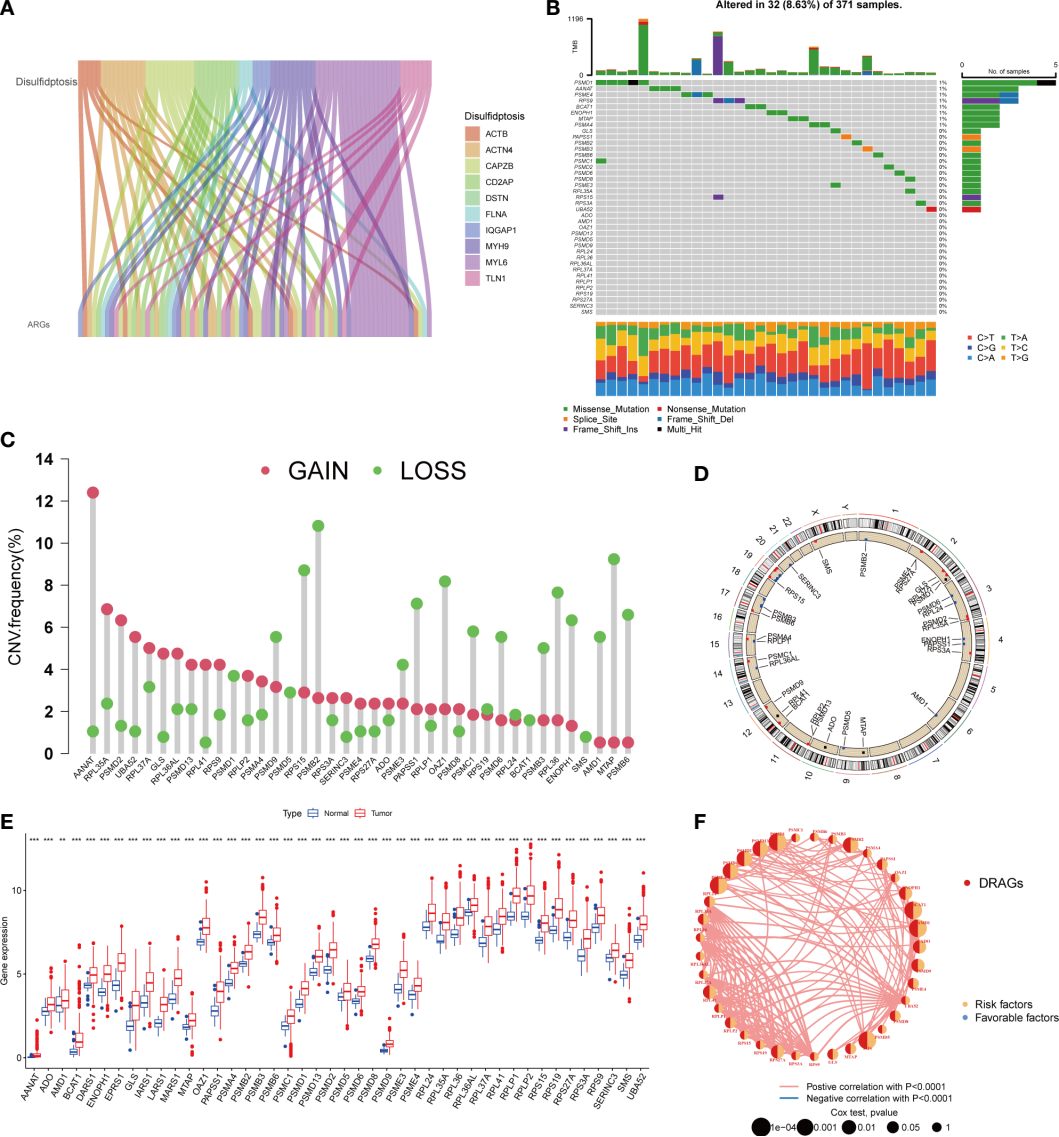
A series of analyses about DRAGs. **(A)** Sankey diagram showing the correlation between DRGs and ARGs. **(B)** Mutation frequencies and mutation types of 39 DRAGs in 371 hepatocellular carcinoma **(HCC)** patients from the TCGA database. **(C)** Frequency of increased and decreased CNV in DRAGs. **(D)** Location of CNVs in DRAGs on 24 chromosomes. Red dots indicate increased copy number, and blue dots indicate decreased copy number. **(E)** Expression of 39 DRAGs between normal and HCC tissues. ** represents P<0.01, *** represents P<0.001. **(F)** Interaction relationship between DRAGs in HCC. The thickness of the connecting line indicates the strength of the correlation effect between genes, pink represents a positive correlation, blue represents a negative correlation.

First, we demonstrated the interactions between ARGs using a Sankey diagram (**Figure 2A**). TMB analysis of DRAGs showed that 32 (8.63%) of the 371 patients had mutations. Among these, the PSMD1 mutation frequency was the highest (4%), followed by AANAT (**Figure 2B**). Next, the somatic copy number variation (CNV) frequency of DRAGs in HCC was evaluated and alterations were found in all gene numbers. Among them, most genes, such as AANAT, RPL35A, EPRS1, PSMD2, and UBA52, had increased CNV frequencies, whereas PSMB2, RPS15, PSMB9, MTAP, AMD1, and RPL36 had decreased CNV (**Figure 2C**). In addition, we showed the location of CNV of DRAGs occurring on chromatin by a ring plot (**Figure 2D**) and found that most DRAGs were located on chromosomes 2, 3, 4, 12, 17, and 19. In addition, we compared the differences in DRAGs expression between HCC and normal tissues and found that most genes, such as AANAT, ADO, and AMD1, were highly expressed in tumor tissues (**Figure 2E**), resulting in a worse patient prognosis (**Figures S1A–P**, **S2A–P**. **S3A–C**). **Figure 2F** shows a positive correlation between DRAGs, which facilitate tumor progression. In addition, most genes were positively correlated with CNV, suggesting that CNV may affect gene expression. Therefore, the analysis of mutations and expression of DRAGs revealed significant differences between HCC and normal tissues, indicating that this gene cluster may play an important role in HCC development.

### 3.2 Construction and prognostic analysis of molecular subtypes of DRAGs in patients with hepatocellular carcinoma (HCC)

**Figure 3:**
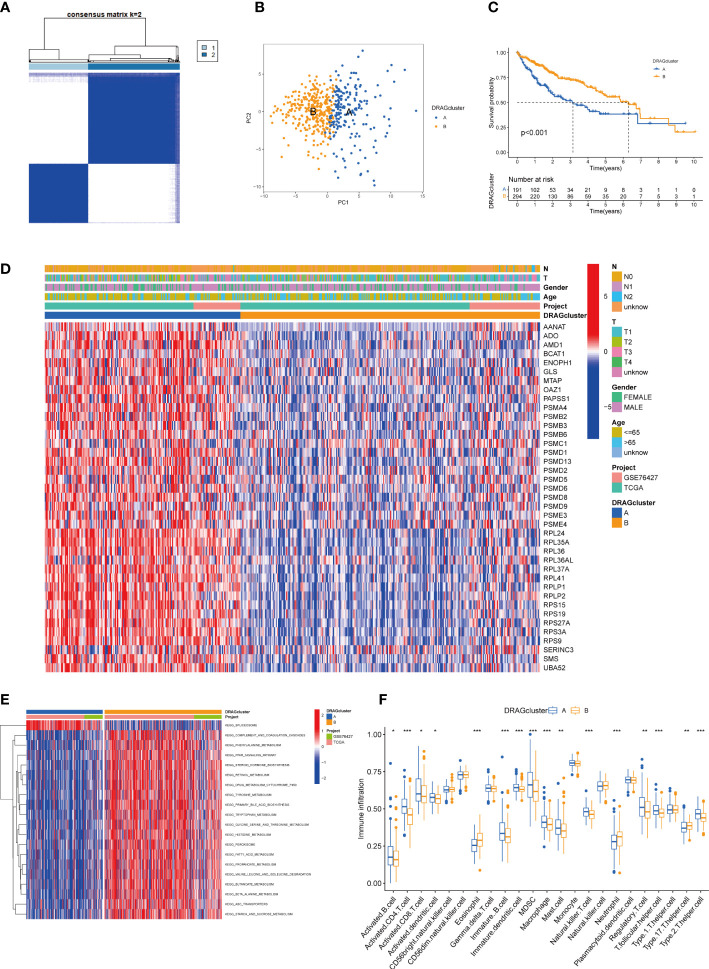
DRAGcluster analysis. **(A)** Consensus matrix diagram defining the relevant regions of the two clusters. **(B)** PCA analysis shows significant differences between the two subtype groups. **(C)** K-M analysis shows the prognostic characteristics of patients in both subgroups. **(D)** Differences in clinicopathological features and expression levels of DRAGs between the two different subtypes. **(E)** GSVA of biological pathways between the two different subtypes, red and blue represent activating and inhibiting pathways, respectively. **(F)** The extent of infiltration of 23 immune cells in HCC subtypes. pca, principal component analysis; gsva, gene set variation analysis. ** represents P<0.01, *** represents P<0.001.

We evaluated HCC subtypes based on differences in the expression of DRAGs and performed a cluster analysis of patients with HCC using TCGA-LIHC and GEO databases (GSE76427). During the cluster analysis of the 486 samples, k=2 was considered the best clustering method to minimize the differences between groups, and patients with HCC were divided into two subtypes: DRAGs cluster A and DRAGs cluster B (**Figure 3A**). PCA showed that the two subtypes could be clearly distinguished, further confirming the accuracy of the typing analysis (**Figure 3B**). In the K-M survival analysis of patients with both subtypes, better survival outcomes were observed in patients with subtype B (**Figure 3C**).

### 3.3 Gene set variation analysis and immune microenvironment analysis of molecular subtypes of DRAGs

First, we plotted a heat map using clinicopathological information, which showed the relationship between sex, age, T and N stages, and the DRAGs cluster; DRAGs were highly expressed in cluster A (**Figure 3D**). GSVA was performed for the two subtypes, and the differences in the enrichment pathways between the two subtypes were compared using KEGG. DRAGs cluster A was highly enriched in the spliceosome pathway, whereas the remaining pathways such as the complement and coagulation cascades, phenylalanine metabolism, and PPAR signaling pathways were highly enriched in cluster B (**Figure 3E**). In addition, we explored the differences in the degree of immune cell infiltration between the two subtypes using ssGSEA. Most of the 23 immune cells were highly infiltrated in cluster A, including activated B cells, CD4 T cells, and CD8 T cells. However, eosinophils, neutrophils, and Th17 cells exhibited high levels of immune cell infiltration in cluster B (**Figure 3F**).

### 3.4 Construction of gene subtypes based on differential genes between molecular subtypes of DRAGs and validation

**Figure 4:**
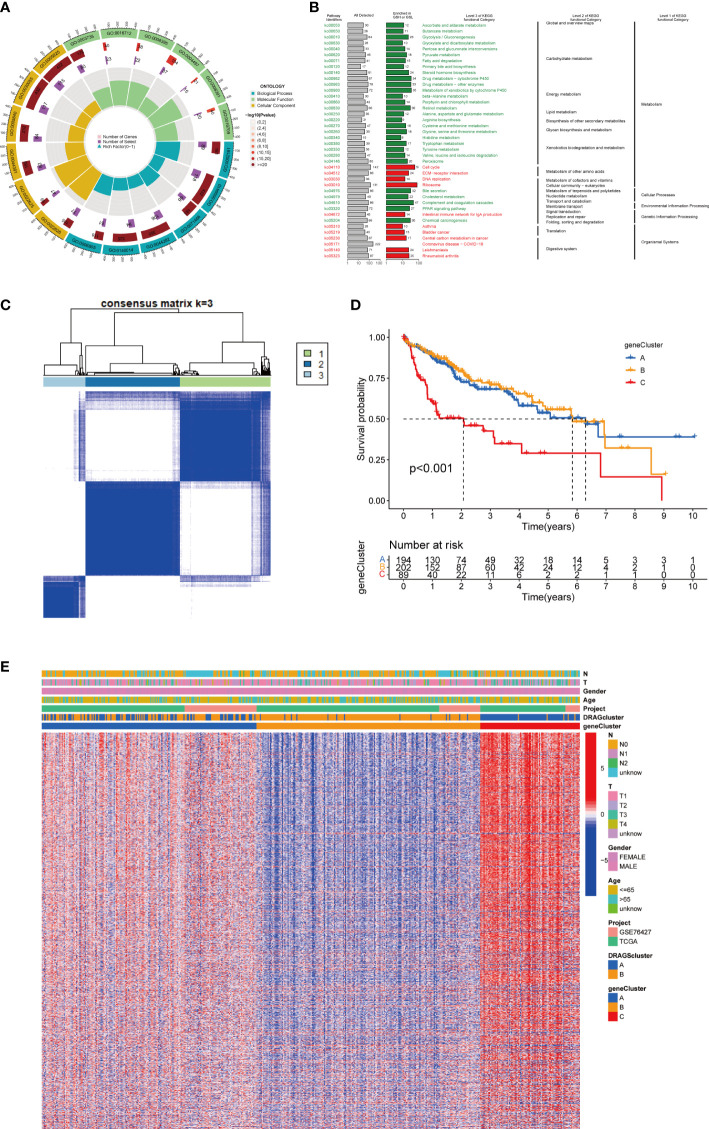
Genecluster analysis. **(A)** GO enrichment analysis of DEGs between two DRAGs subtypes. The red part of the graph represents the number of enriched genes, the redder the color means the more significant gene enrichment; the purple part represents the number of enriched differential genes. The bar graph represents the proportion of genes. **(B)** KEGG enrichment analysis of DEGs between two DRAGs subtypes. **(C)** Consensus matrix plot defining the three cluster-related regions. **(D)** Kaplan-Meier curves for the three gene subtypes. **(E)** Relationship between the three gene subtypes and clinicopathological features.

First, to explore the potential biological behaviors of tumor cells, we screened a total of 2008 differential genes between DRAGs cluster A and cluster B by the “limma” package in R Studio, with the screening condition: log|FC|≥0.585, corrected p-value<0.05. Next, using GO functional enrichment analysis, we found that the differential genes were mainly enriched in the BP functional set, such as cytoplasmic translation and xenobiotic metabolic processes, and were related to the CC functional set, such as the cytosolic ribosome and cytosolic large ribosomal subunit, which play important roles in HCC progression (**Figure 4A**; **Table S3**). KEGG pathway enrichment analysis of the differentially expressed genes suggested that the main pathways involved were Glycolysis/Gluconeogenesis and Glycine, serine, and threonine metabolism (**Figure 4B**; **Table S4**). These results suggest that DRAGs may act by affect the metabolism of HCC cells.

**Figure 5:**
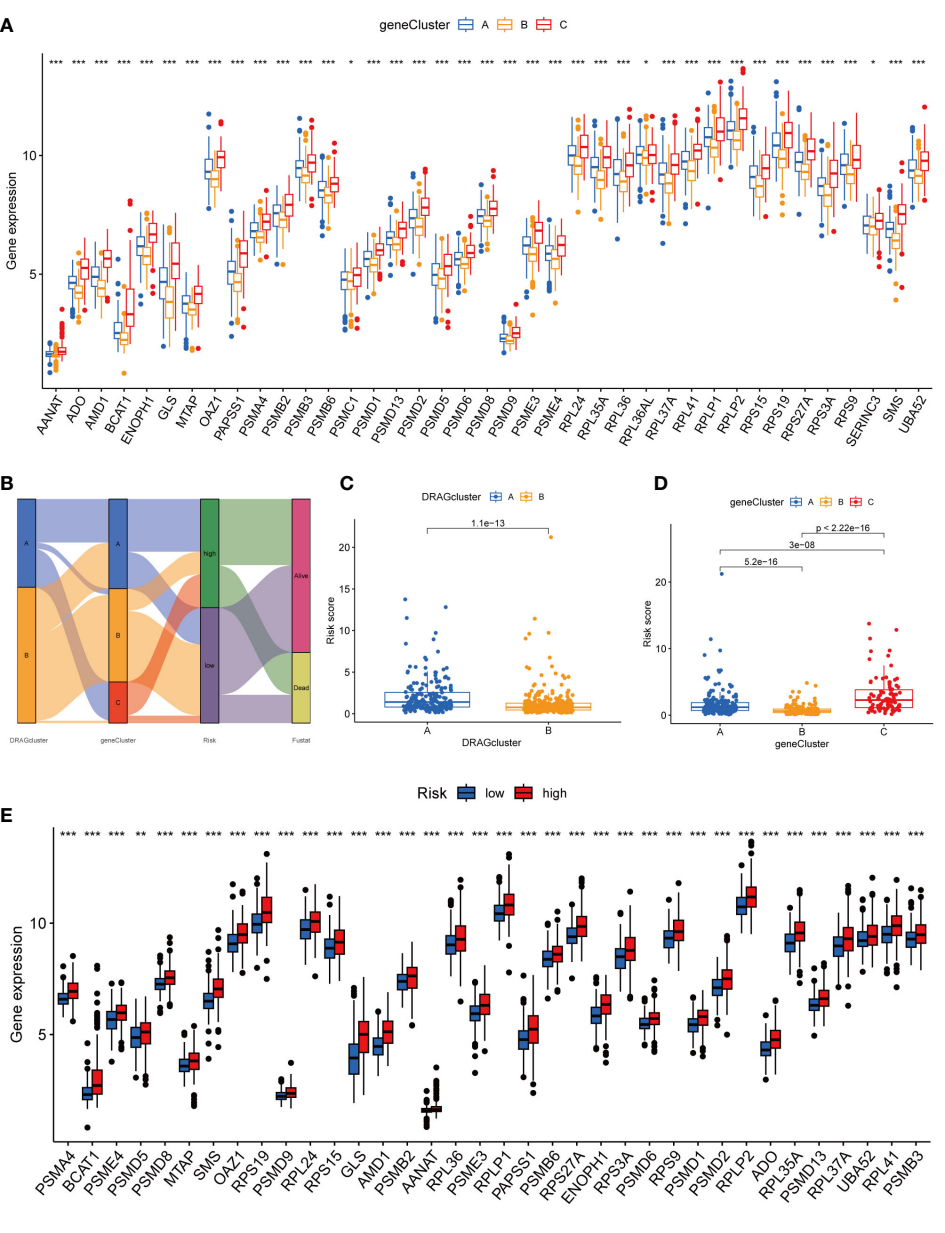
Genecluster and its risk prognosis signature. **(A)** Differential expression of 39 DRAGs in the three gene subtypes. **(B)** Sankey diagram of different HCC subtypes and survival outcomes. **(C)** Differences in risk scores among different DRAGs subtypes. **(D)** Differences in risk scores among different gene subtypes. **(E)** Differences in expression of 39 DRAGs in high and low-risk groups. * represents P<0.05, ** represents P<0.01, *** represents P<0.001.

We then performed univariate Cox regression analysis to obtain 1139 genes with prognostic value for subsequent analyses. To further validate this regulatory mechanism, the samples were typed again according to the 1139 prognostic genes, and the “ConsensusClusterPlus” algorithm in R language was applied to obtain a clustering map with k=3 as the best clustering method for the samples (**Figure 4C**), and three gene subtypes were obtained, named gene cluster A, B and C. The cumulative density function (CDF) curves verified the clustering accuracy (**Figure S3D**). K-M analysis suggested that patients with gene cluster C had the worst prognosis, whereas those with gene cluster B had a higher survival rate (p< 0.001) (**Figure 4D**). A heat map of the clinicopathological features showed that gene cluster C mainly corresponded to DRAGs cluster B, and that patients with both subtypes had the worst prognosis (**Figure 4E**). In addition, analysis of the expression of DRAGs in patients with the three gene subtypes revealed that the expression of DRAGs decreased sequentially in gene clusters C, A, and B, with statistically significant differences (**Figure 5A**).

### 3.5 Construction and validation of risk prognostic signature

First, we constructed a prognostic signature for DRAGs from the differential genes among the three gene subtypes based on significant gene data obtained from multifactorial Cox regression analysis using LASSO regression analysis to avoid overfitting (**Figures S2B, C**). Six genes were included: CFHR3, GPX7, FAM83D, CD8A, EPO and MSC, and the risk score formula was: Risk score = (-0.1072*expression of CFHR3) + (0.2380*expression of GPX7) + (0.2382*expression of FAM83D) + (-0.4009*expression of CD8A) + (0.1751*EPO expression) + (0.2613*MSC expression). Sankey plots indicated a consistent relationship between the two molecular subtypes of DRAGs, the three gene subtypes, high- and low-risk groups for prognostic signatures, and patient survival (**Figure 5B**). Second, we evaluated the relationship between the three gene subtypes and risk scores and observed that subtype A exhibited a higher risk score than DRAGs cluster B. More importantly, gene cluster B had the lowest risk score, whereas subtype C had the highest score, which is consistent with the data from the survival analysis described above (**Figures 5C, D**). In addition, DRAGs were highly expressed in the high-risk group as potential oncogenic factors that are highly expressed in HCC tissues (**Figure 5E**).

**Figure 6:**
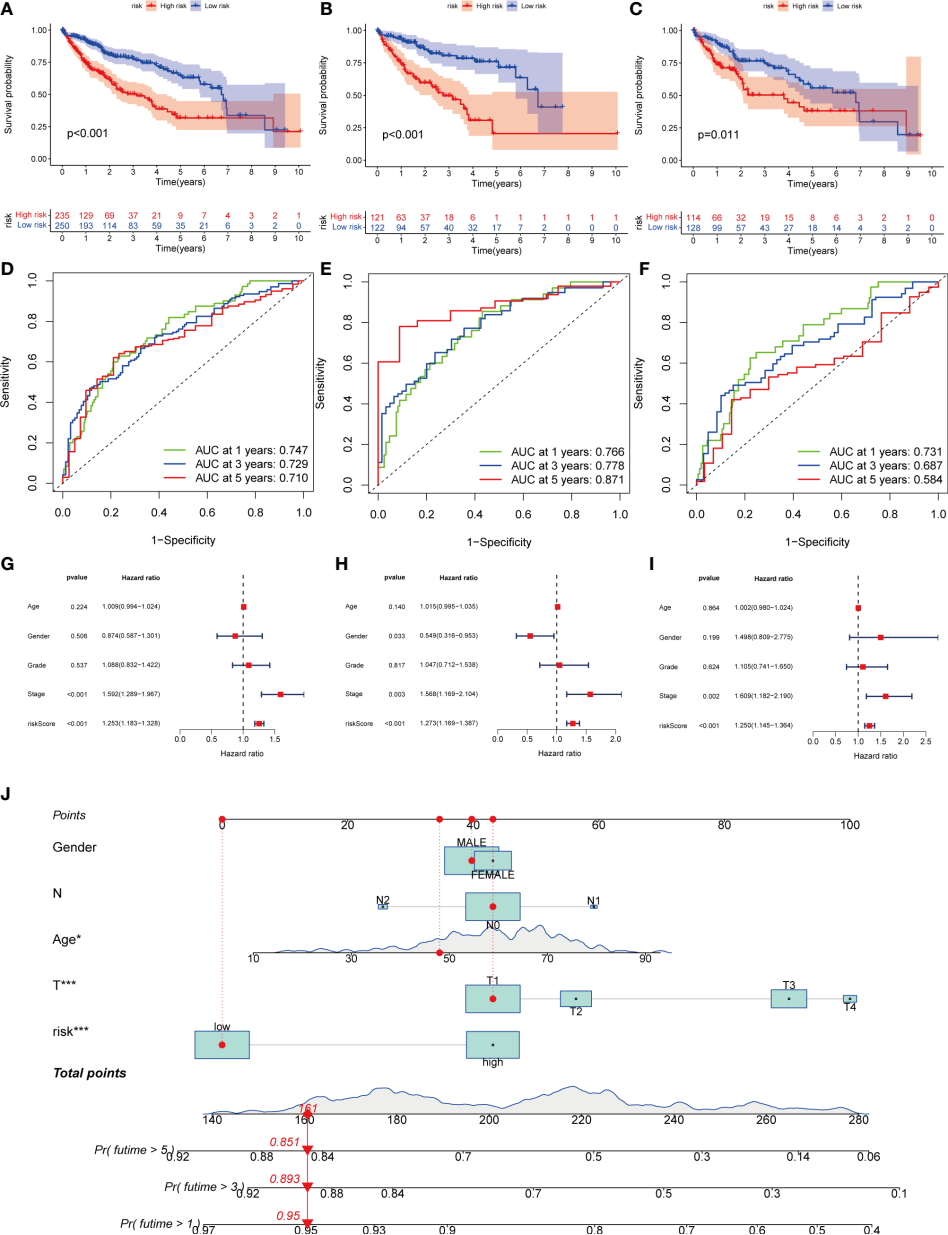
Kaplan-Meier analysis, ROC curves, and nomogram of risk groups. **(A)** Kaplan-Meier analysis of the Recurrence free survival (RFS) of all-risk and low-risk groups in the training group **(B)** Kaplan-Meier analysis of the RFS of high-risk and low-risk groups in the training group. **(C)** Kaplan-Meier analysis of the RFS of high-risk and low-risk groups in the training group. **(D)** ROC curves predict 1-, 3-, and 5-year survival of all group patients. **(E)** ROC curves predicting 1-, 3-, and 5-year survival rates for patients in the train group. **(F)** ROC curves predicting 1-, 3-, and 5-year survival rates for test group patients. **(G)** The multivariate Cox regression analysis of clinical characteristics and risk score in all groups. **(H)** The multivariate Cox regression analysis of clinical characteristics and risk score in train group. **(I)** The multivariate Cox regression analysis of clinical characteristics and risk score in test group. **(J)** Construction of a nomogram based on clinical characteristics and risk score for prognostic signature. **(J)** ROC, receiver operating characteristic. * represents P<0.05, ** represents P<0.01, *** represents P<0.001.

Next, we divided the patients from TCGA and GEO databases equally into training and test groups at a 1:1 ratio. The patients were divided into high- and low-risk groups based on the median risk score. K-M analysis suggested that patients with gene cluster C had the worst prognosis, whereas those with gene cluster B had a higher survival rate (P< 0.01) (**Figures 6A–C**). ROC analysis of all patients with HCC according to the risk-prognosis signature showed that the areas under the curve (AUC) were 0.753, 0.708, and 0.666 at 1, 3, and 5 years, respectively (**Figure 6D**). In the training group, the one-, three-, and 5-year AUCs were 0.814, 0.757, and 0.804, respectively (**Figure 6E**), whereas those in the test group were 0.692, 0.661, and 0.575, respectively, confirming the diagnostic power of the signature (**Figure 6F**). Subsequently, we determined the prognostic value of the tumor stage and risk score in the three groups using univariate Cox regression analysis. Multivariate Cox regression analysis suggested that the risk score was an independent prognostic factor in all groups (**Figures 6G–I**).

### 3.6 Creation of nomogram

Owing to the limitations of the scoring system alone in clinical applications, we combined scores and clinical information to create a nomogram to predict the survival time of patients at one, three- and 5-years. Both the T stage and risk score were independent prognostic factors (**Figure 6J**). The calibration chart further confirmed the accuracy of the signature (**Figure S3G**).

### 3.7 Assessment of tumor immune microenvironment and biological characteristics among different risk groups

**Figure 7:**
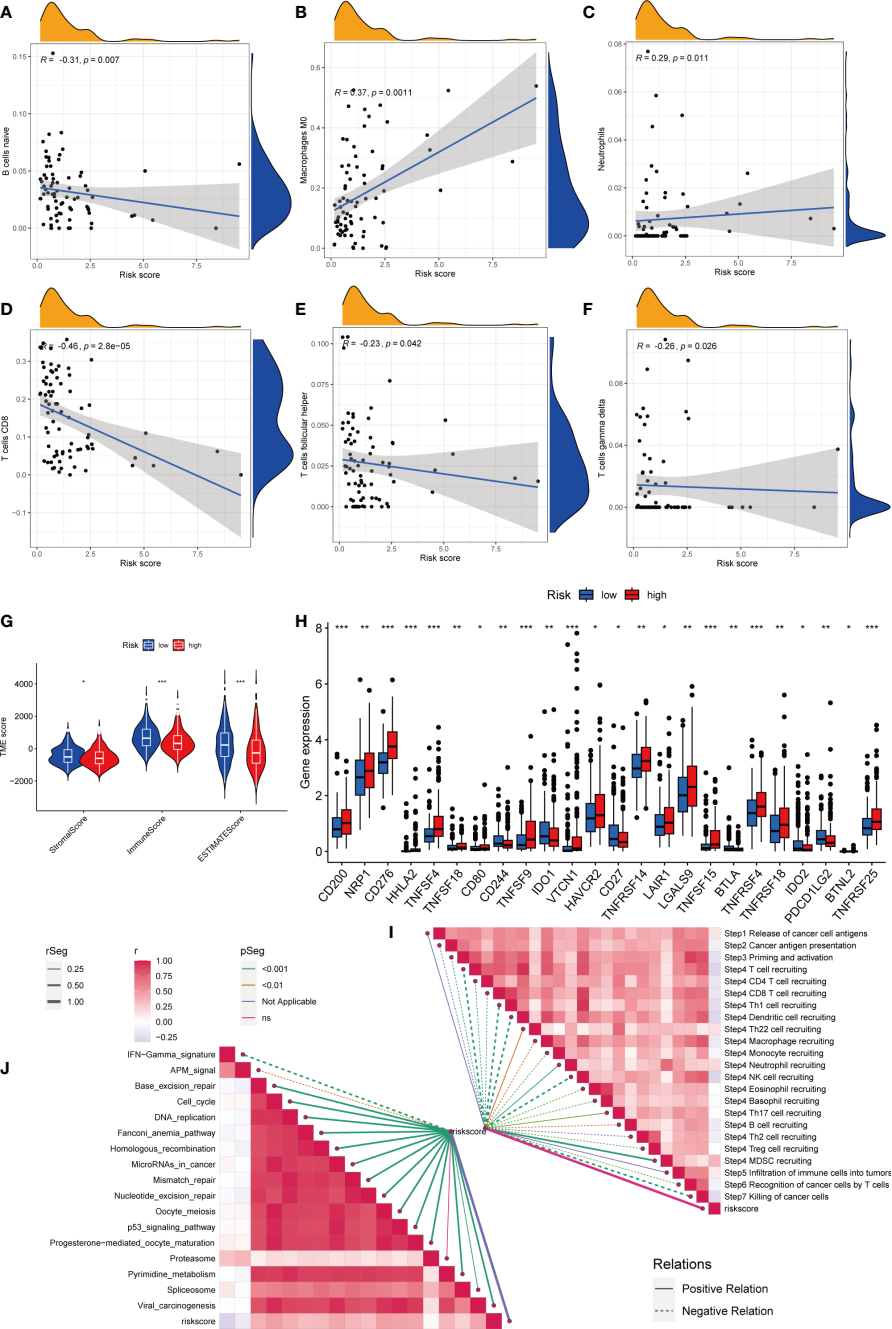
Risk score and analysis of infiltrating immune cells in HCC. **(A–F)** Correlation of Risk score with immune cells. **(G)** Correlation of risk score with an immune score, stromal score, and tumor purity. **(H)** Differences in expression of immune checkpoints in high- and low-risk groups. **(I)** Correlation between Risk score and enrichment of immunotherapy-related pathways. **(J)** Correlation between Risk score and tumor immune response process. * represents P<0.05, ** represents P<0.01, *** represents P<0.001.

First, we assessed the association between risk scores and immune cells using Spearman’s correlation analysis, and the results were visualized using scatter plots. Naive B cells, CD8 + T cells, and follicular helper T cells negatively correlated with risk scores, whereas M0 macrophages and neutrophils positively correlated with risk scores (**Figures 7A–F**). At the same time, the proportion of HCC-infiltrating immune cells was visualized for each sample (**Figure S3H**). In addition, the TME scores indicated that the low-risk group had higher stromal and immune scores, and higher tumor purity (**Figure 7G**). Second, the differences in immune checkpoint expression suggested that most immune checkpoint molecules such as CD200, NRP1, and CD276 were highly expressed in the high-risk group. Interestingly, CD244, CD27, IDO2, and PDCD1 were highly expressed in the low-risk group (**Figure 7H**). Suggesting that immune checkpoints are involved in tumor progression and are promising candidates for application in the high-risk group to guide immunotherapy. Finally, based on Hu et al., we obtained the steps of the cancer immunity cycle dataset and enrichment scores of the immunotherapy-predicted pathway dataset (22). “GSVA” and “ggcor” package was used to construct risk score correlation with the datasets. As suggested by previous results, the risk score was mostly negatively correlated with the steps of the cancer immunity cycle, including CD4 + T cell, CD8 + T cell, and B-cell recruitment (**Figure 7I**; **Table S5**). Interestingly, the risk score positively correlated with most of the enrichment scores of the immunotherapy-predicted pathways, except for IFN-γ and APM signaling (**Figure 7J**; **Table S6**).

### 3.8 Relationship between risk scores and TMB, MSI, and CSC indices

**Figure 8:**
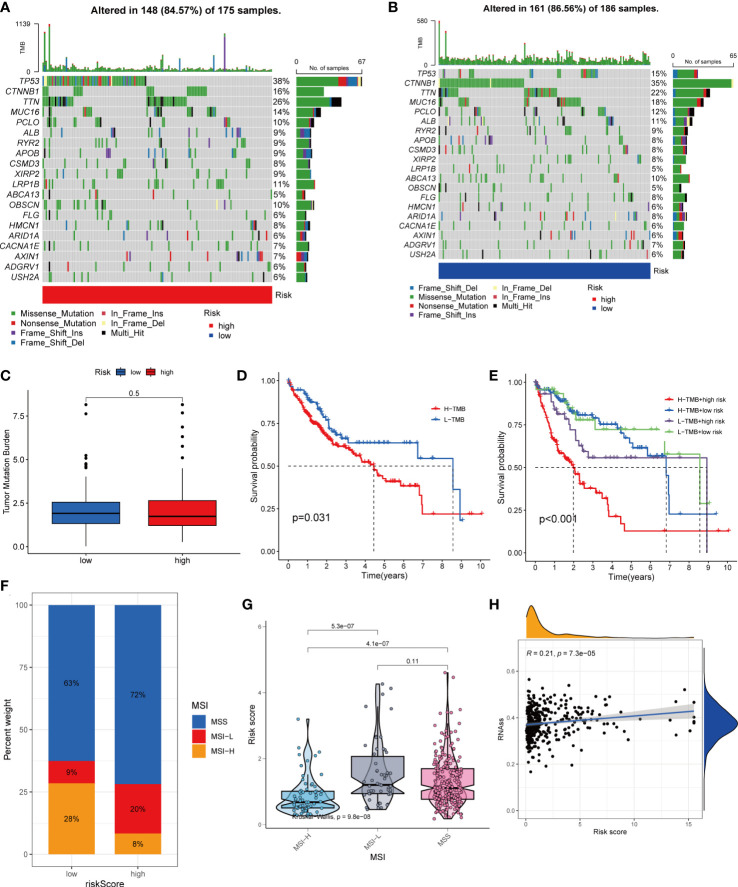
Comprehensive analysis of the risk score in HCC. **(A, B)** Waterfall plots of somatic mutation frequencies and mutation types between different risk groups. Each column represents an individual patient. The bar above each column shows the TMB, and the number on the right side indicates the mutation frequency of each gene. The bar on the right shows the proportion of each mutation type. **(C)** Differences in TMB across risk groups. **(D)** Survival differences between high and low TMB groups in HCC. **(E)** Survival differences between patients with combined TMB and risk score assessment in HCC. **(F, G)** Relationship between Risk score and MSI. **(H)** Relationship between Risk score and CSCs.

HCC development is influenced by multiple complex factors including TMB, MSI, and CSCs. Therefore, it is crucial to explore the relationships between prognostic signatures and these factors. We included 361 HCC patients with complete mutation information from TCGA database. The 20 genes with the highest mutation frequencies were selected for visualization using waterfall plots. Comparative analysis of the high- and low-risk groups showed that TMB occurred in 84.57% of the patients in the high-risk group, with the most significant mutation in TP53 (38%; **Figure 8A**). TMB occurred in 86.56% of samples in the low-risk group, with the most significant mutation in CTNNB1 (35%; **Figure 8B**). The difference in TMB between the high- and low-risk groups was not statistically significant (P=0.5) (**Figure 8C**). Notably, survival analysis suggested a better prognosis in the low-TMB group (P<0.05) (**Figure 8D**). In addition, by combining the risk score and TMB from the prognostic signature, survival analysis showed statistically significant survival among the four groups (P<0.001) (**Figure 8E**). In conclusion, although the difference in TMB between the high- and low-risk groups was not statistically significant, TMB combined with the risk score was a better predictor of OS.

For oncology patients, it has been shown that, the higher the MSI, the higher the potential for selecting immunotherapy (23). MSI has been suggested that MSI is a biomarker for determining the immune checkpoint therapy response (24). Our analysis of patients with HCC showed that the MSI-H group had a lower risk score than the MSS and MSI-L groups(P<0.01; **Figures 8F, G**).

In addition, we assessed the association between tumor cell stemness (CSC) and the signature risk score, and correlation analysis showed a positive correlation between CSC and the risk score (R = 0.21, p< 0.001). These results suggest that patients with high-risk HCC have more significant tumor stem cell characteristics, a lower degree of cell differentiation, and a higher degree of malignancy (**Figure 8H**).

### 3.9 Expression and immune infiltration characteristics of 6 genes in the signature

**Figure 9:**
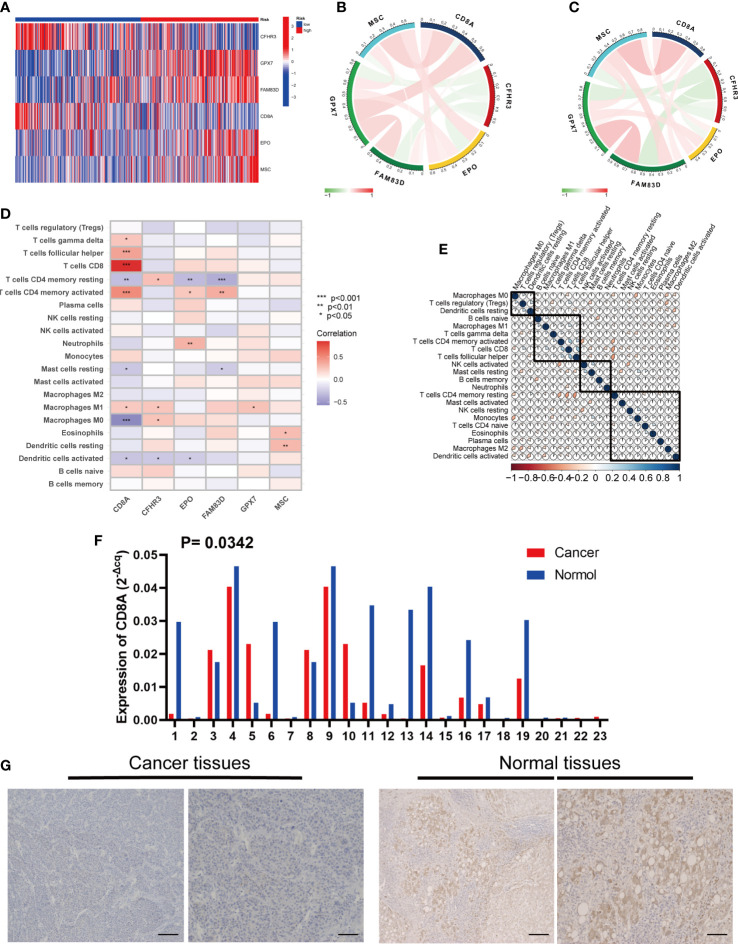
Six risk model genes interaction and core gene verification. **(A)** Expression of the six genes in the risk signature in the high and low risk groups. **(B)** Correlation of the six genes in the train group. **(C)** Correlation of the six genes in the test group. **(D)** Correlation between the level of immune cell infiltration and the six genes in the risk signature. **(E)** Correlation clustering of HCC infiltrating immune cells. **(F)** qRT-PCR of CD8A expression differences in HCC tissues and normal tissues. **(G)** Normal and cancer images of CD8A expression in liver tissues (100× and 200×) detected by IHC staining. * represents P<0.05, ** represents P<0.01, *** represents P<0.001.

First, we assessed differences in the expression of the six genes between the high- and low-risk groups (**Figure 9A**). We explored the interactions among the six genes in the training group. CFHR3 expression was significantly and negatively correlated with the remaining five genes, whereas GPX7, FAM83D, CD8A, EPO, and MSC expression were positively correlated (**Figure 9B**). The results of the test group were consistent (**Figure 9C**).

In addition, we evaluated the relationship between the six genes in the signature and immune cells, and found that CD8A and CFHR3 were strongly correlated with immune cells (**Figure 9D**).

**Figure 10:**
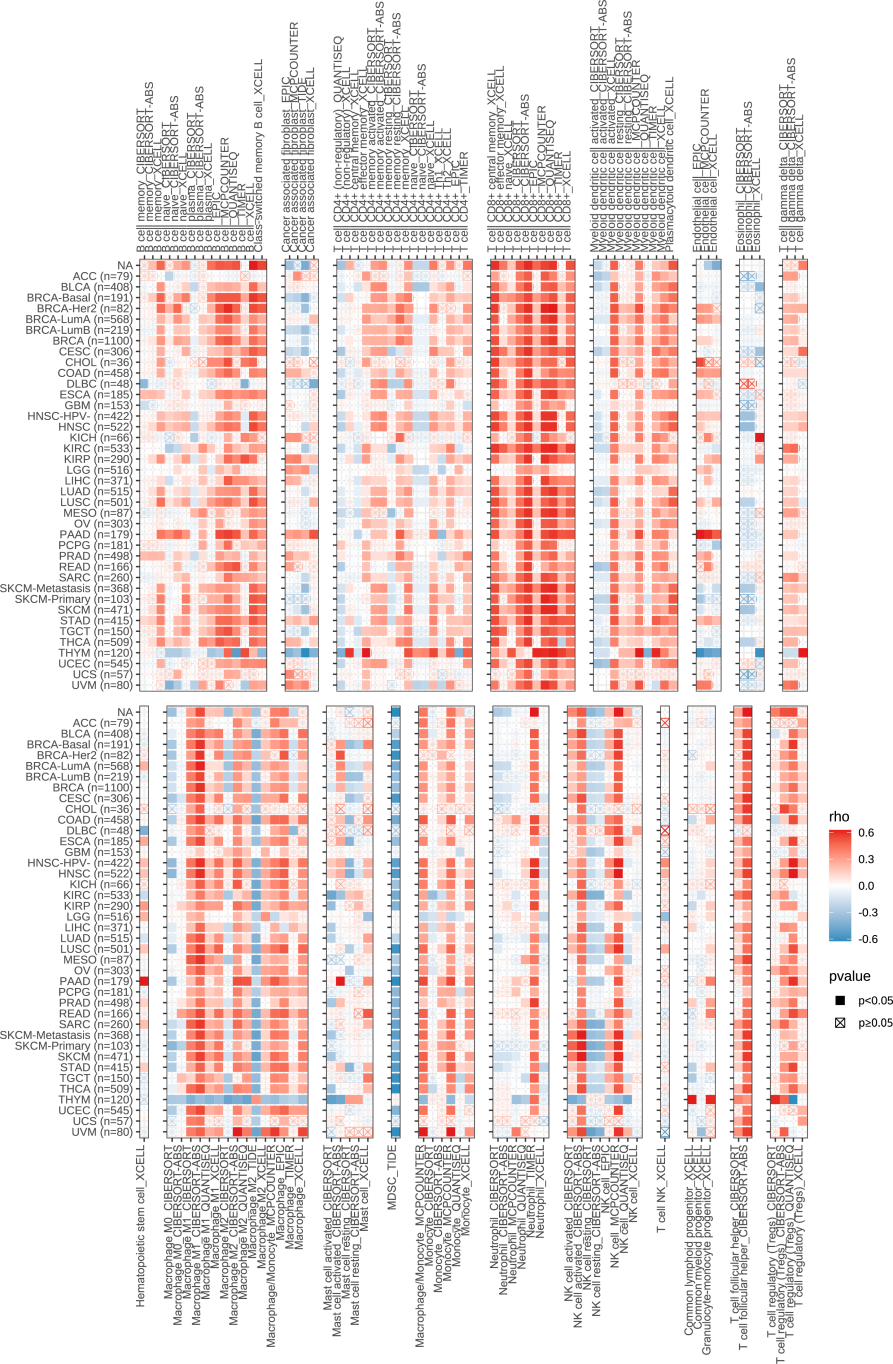
Correlation between the expression of CD8A and the level of infiltration of various immune cells in pan-cancer.

Next, we performed a correlation clustering analysis of HCC-infiltrating immune cells (**Figure 9E**). As expected, risk score-associated immune cells such as naïve B cells, CD8 T cells, and follicular helper T cells were clustered in a correlation set. Because the strongest correlation between these six genes and immune cells was observed, CD8A attracted our attention. Using qRT-PCR (n=23) and IHC (n=20), the mRNA and protein expression levels of CD8A were verified in HCC tissues. CD8A expression was lower in HCC tissues than in normal tissues (**Figures 9F, G**; **Table S7**). Based on pan-cancer analysis, we found that CD8A was positively correlated with most tumor-infiltrating cells, especially B cells, dendritic cells, CD8+ T cells, macrophages, regulatory T cells (Tregs), and follicular helper T cells (**Figure 10**).

### 3.10 Drug sensitivity analysis

**Figure 11:**
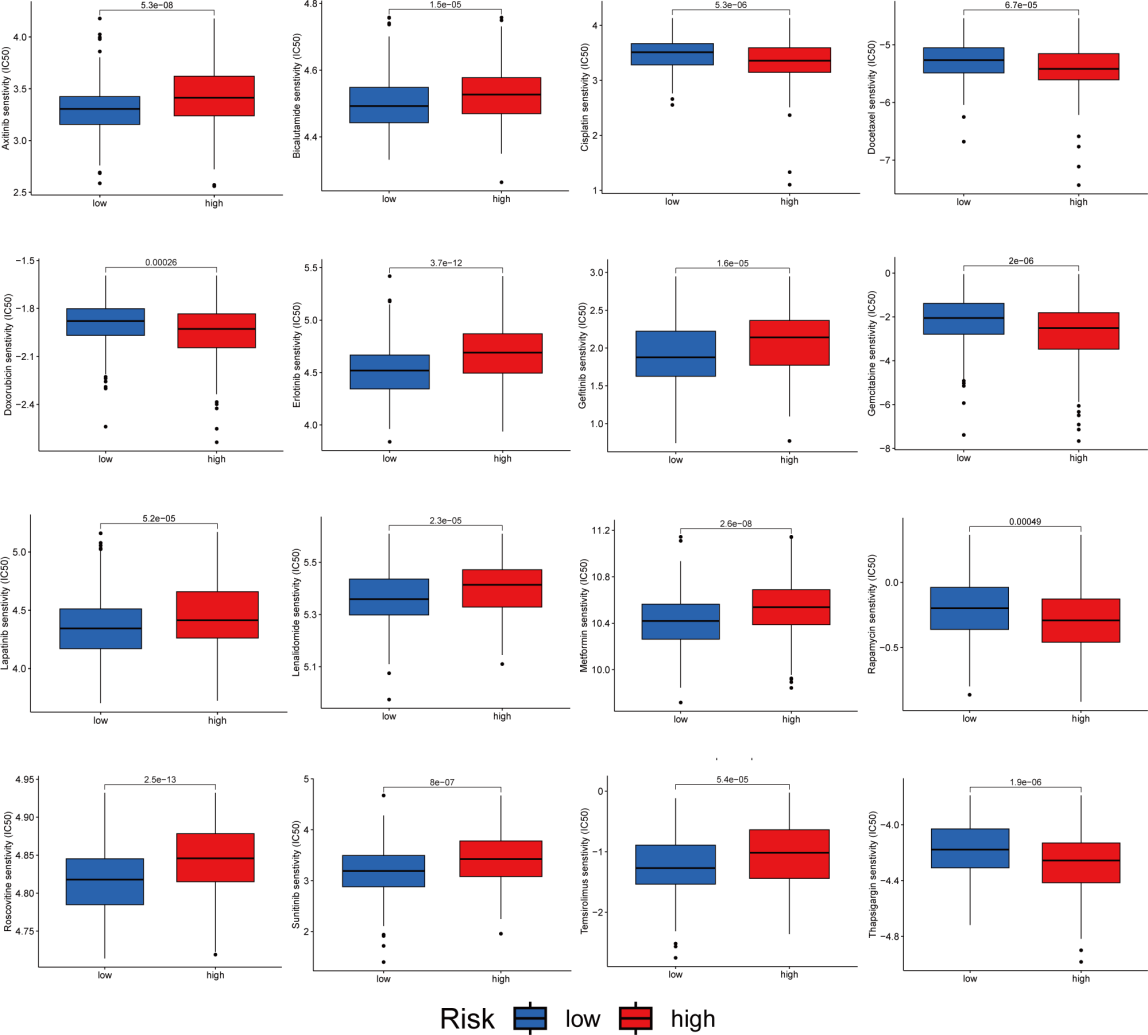
Relationship between patients with two risk score subtypes and drug sensitivity.

We analyzed the sensitivity of patients in the high- and low-risk groups to chemotherapeutic agents commonly used to treat HCC. We and found that patients in the low-risk group were more sensitive to drugs such as Axitinib, Bicalutamide and Erlotinib. However, the patients in the high-risk group were more sensitive to Cisplatin, Docetaxel and Doxorubicin (**Figure 11**). In conclusion, there were significant differences in drug sensitivity among different HCC subtypes, providing a direction for personalized treatment of HCC.

## 4 Discussion

HCC is a highly heterogeneous tumor, particularly in terms of genomics, transcriptomics, proteomics and metabolomics (25). Disulfidoptosis is a recently identified rapid mode of programmed cell death caused by the intracellular accumulation of excess cystine resulting from disulfide stress, which usually occurs under conditions of glucose starvation (26). Some studies have demonstrated the potential of targeting disulfidoptosis in tumor therapy (4, 6). In recent years, disulfide proteomics has been proposed. Protein synthesis and degradation are regulated by cysteine redox state (27). The liver is the main organ involved in amino acid metabolism, and these processes have been shown to be closely associated with HCC (28). Metabolic variants accelerate tumor progression. The reprogramming of amino acid metabolism is an important metabolic variant. Many metabolism-related genes have been shown to be effective prognostic markers of HCC. However, studies regarding the role of DRAGs in HCC are lacking.

In the present study, we investigated the correlation between DRAGs and HCC. Surprisingly, these genes were not significantly mutated in the HCC cells. However, this did not diminish their importance as their differential expression in HCC and normal tissues was more important. Subsequently, we classified patients with HCC into two different cell subtypes based on the DRAGs. Patients with DRAG subtype A showed poorer pathological staging and overall survival than those with DRAGs cluster B. In addition, there were significant differences in gene expression and metabolic pathway enrichment between subtypes. Immune cell infiltration into the TME also differed significantly between the two subtypes. We obtained three gene subtypes by consensus clustering of patients with HCC based on differentially expressed genes between the cell subtypes of the two DRAGs. These results suggested that DRAGs are promising predictors of HCC prognosis and sensitivity to immunotherapy. Additionally, we screened six genes with prognostic value using multifactorial Cox regression analysis to construct a risk prognostic signature. Significant differences in the clinicopathological characteristics, prognosis, TME, immune checkpoint expression, TMB, MSI, CSC index, and drug sensitivity were observed between the high- and low-risk groups. In addition, we established a nomogram for better clinical applicability and found that the signature had high diagnostic value based on ROC curves. Therefore, accurate identification of the molecular subtypes of HCC under conditions of tumor heterogeneity and construction of prognostic signatures are crucial for the development of new targeted therapeutic regimens for personalized precision medicine (29).

From the perspective of prognostic value, among the six genes used to construct the signature, the protective values of CFHR3 and CD8A have been demonstrated in a variety of tumors, whereas GPX7, FAM83D, EPO, and MSC are involved in tumor progression. Several factors affect the expression of prognosis-related genes in patients with HCC. CD8A, a glycoprotein mainly located on the surface of T lymphocytes, is downregulated in HCC and acts as a barrier against anti-tumor immunity (30). As the strongest correlation among the six genes with tumor immune cells, CD8A is a potential hub gene that connects disulfidoptosis and immune reprogramming. To avoid the influence of the different ethnic structures of the database and our country, we needed to verify the low expression of CD8A in HCC before conducting follow-up research. As expected, IHC and RT-qPCR results were consistent with the database analysis’s. GPX7, a glutathione peroxidase, is overexpressed in HCC tissues and has prognostic and diagnostic value (31). Overall, these genes are closely associated with tumorigenesis and development.

Multiple factors influence HCC prognosis due to tumor heterogeneity. The highest percentage of TP53 mutations was found in the high-risk group (up to 38%) compared with only 15% in the low-risk group. Mutations in the tumor suppressor TP53 are the most common in HCC and often lead to poor prognosis and reduced immune responses in patients with HCC (13, 32). In addition, CTNNB1 mutations were more frequent in the low-risk groups. In most cases, CTNNB1 mutations are associated with immune rejection and-β-catenin pathway (33). Therefore, the poor prognosis of patients with HCC in the high-risk group may be attributed to the high number of TP53 mutations. Therefore, CTNNB1 should be considered as a potential therapeutic target in low-risk patients.

The liver contains many immune cells and is the largest organ in the body. Immune cells in the TME play an important role in promoting tumor growth and inhibiting cancer progression, and immunotherapy has become a popular topic in tumor treatment. We combined Spearman’s correlation analysis of risk scores and immune cells with activity analysis of the anti-cancer response process and found that CD8+ T cells showed low infiltration, M0 macrophages and neutrophils showed high infiltration, and neutrophils were more actively recruited in the high-risk group. Previous studies have shown that M0 macrophages from the bone marrow rest before cell differentiation, but M0 macrophages are strongly associated with poor patient prognosis in gliomas (34, 35), and M0 macrophage infiltration is significantly increased in patients with high-risk endometrial cancer (36). In addition, neutrophils play an important role in the tumor immunosuppressive microenvironment, promote tumor progression, and are potential therapeutic targets for HCC (37). However, T and Th1 cell recruitment was higher in the low-risk group. In addition, regarding immune-related enrichment pathways, our study found that IFN-γ and APM signaling were significantly enriched in the low-risk group. It has been shown that the anti-tumor effect of IFN-γ can be promoted by enhancing the immune response of Th1 cells in tumor immunotherapy. Additionally, as an anti-tumor factor, IFN-γ plays an immunosuppressive role in tumors such as melanoma and lung cancer by enhancing the immune response of T lymphocytes (38). Our study showed that patients in the high-risk group had lower immune scores and a stronger immunosuppressive microenvironment, promoting tumorigenesis and metastasis and leading to a worse prognosis. However, higher immune checkpoint expression indicates that the high-risk group is more likely to benefit from future immunotherapies targeting immune checkpoints.

Our study has several limitations. First, all patient information was obtained from public databases, mainly Western case data, which lack representative prospective data. Second, the sample had limited clinical information and lacked information on important factors for determining the prognosis of patients with HCC, such as methemoglobin levels, ascites, portal hypertension, and postoperative complications. In future studies, we aim to recruit patients at our hospital who meet the inclusion criteria to further verify the authenticity and validity of the results.

## 5 Conclusion

In brief, we developed a prognostic signature for HCC based on the molecular typing of DRAGs to assist in predicting HCC progression and provide a reference value for targeted therapy and immunotherapy for HCC.

## Data availability statement

The data used to support the findings of this study are available from the corresponding author on request.

## Ethics statement

The studies involving human participants were reviewed and approved by Biomedical Ethics Committee of the First Affiliated Hospital of Chongqing Medical University, The First Affiliated Hospital of Chongqing Medical University. The patients/participants provided their written informed consent to participate in this study.

## Author contributions

XC conceived the project. XC and ZW contributed to data acquisition, analysis and interpretation, and manuscript writing. YW conducted the experiments. YHL and YGL modified the manuscript. All authors contributed to the article and approved the submitted version.

## Supplementary material

Supplementary Figure 1
**(A–P)** The Kaplan–Meier curves for survival status and survival time in the high and low expression groups of DRAGs. **(A)** AANAT. **(B)** ADO. **(C)** AMD1. **(D)** BCAT1. **(E)** ENOPH1. **(F)** GLS. **(G)** MTAP. **(H)** OAZ1. **(I)** PAPSS1. **(J)** PSMA1. **(K)** PSMB2. **(L)** PSMB3. **(M)** PSMB6. **(N)** PSMD1. **(O)** PSMD2. **(P)** PSMD5.

Supplementary Figure 2 **(A-P)** The Kaplan–Meier curves for survival status and survival time in the high and low expression groups of DRAGs. **(A)** PSMD6. **(B)** PSMD8. **(C)** PSMD9. **(D)** PSMD13. **(E)** PSME3. **(F)** PSME4. **(G)** RPL24. **(H)** RPL35A. **(I)** RPL36. **(J)** RPL37A. **(K)** RPL41. **(L)** RPLP2. **(M)** RPS3A. **(N)** RPS9. **(O)** RPS15. **(P)** RPS27A.

Supplementary Figure 3
**(A-E)** The Kaplan–Meier curves for survival status and survival time in the high and low expression groups of DRAGs. **(A)** SERINC3. **(B)** SMS. **(C)** UBA52. **(D)** Cumulative density function (CDF) curves validate the clustering accuracy. **(E, F)** LASSO regression analysis and consistency test. **(G)** Calibration plot of the nomogram. **(H)** Histogram of the percentage of infiltrating immune cells in patients with liver cancer.
