# Supplementary figures and images for: Typing and modeling of hepatocellular carcinoma based on disulfidptosis-related amino acid metabolism genes for predicting prognosis and guiding individualized treatment
Source: Front Oncol. 2023 Aug 11;13:1204335. doi: 10.3389/fonc.2023.1204335 (PMC10454915; doi:10.3389/fonc.2023.1204335)

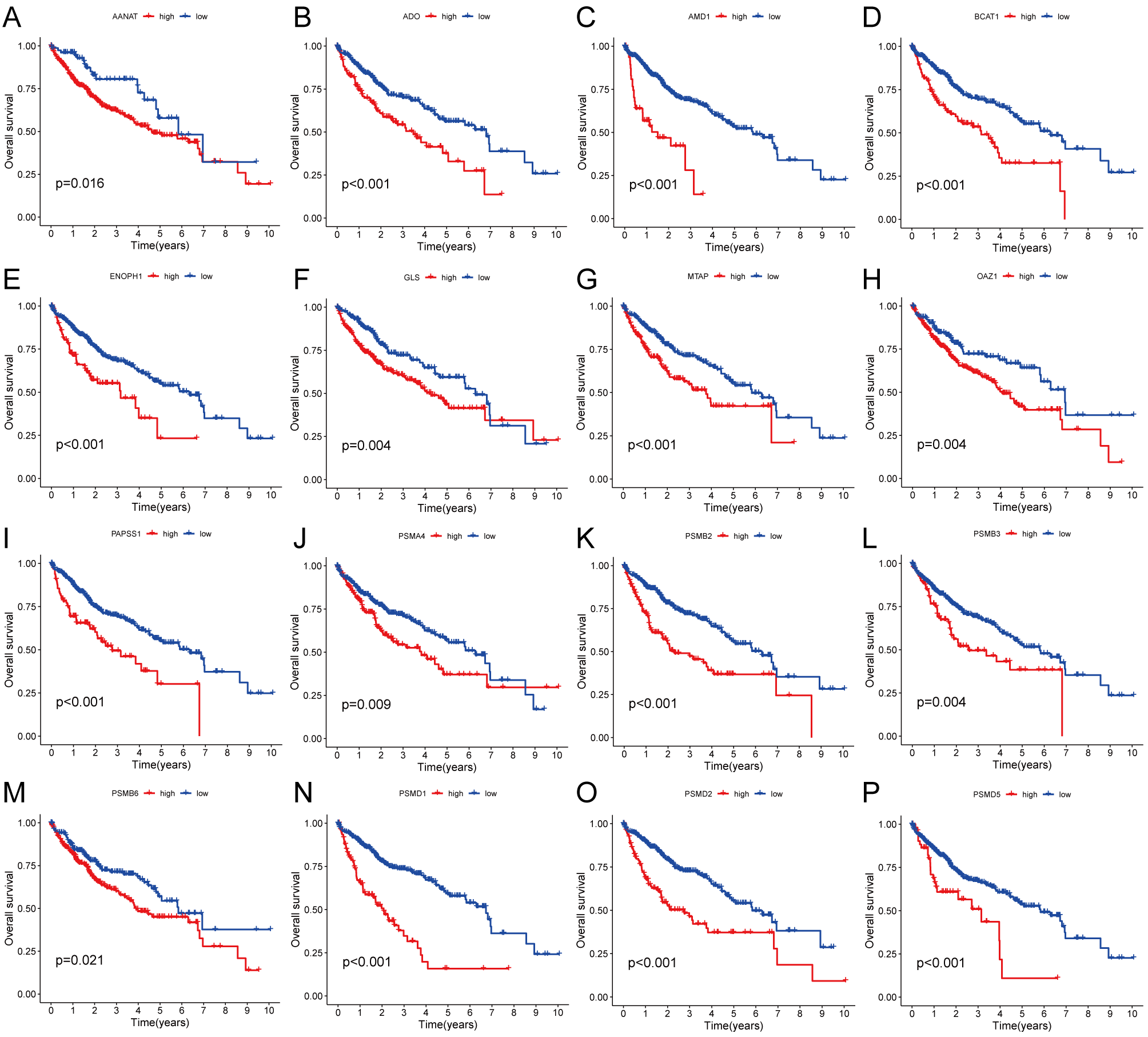

Supplement: Supplementary Figure 1 — (A–P) The Kaplan–Meier curves for survival status and survival time in the high and low expression groups of DRAGs. (A) AANAT. (B) ADO. (C) AMD1. (D) BCAT1. (E) ENOPH1. (F) GLS. (G) MTAP. (H) OAZ1. (I) PAPSS1. (J) PSMA1. (K) PSMB2. (L) PSMB3. (M) PSMB6. (N) PSMD1. (O) PSMD2. (P) PSMD5. [file Image_1.tif]

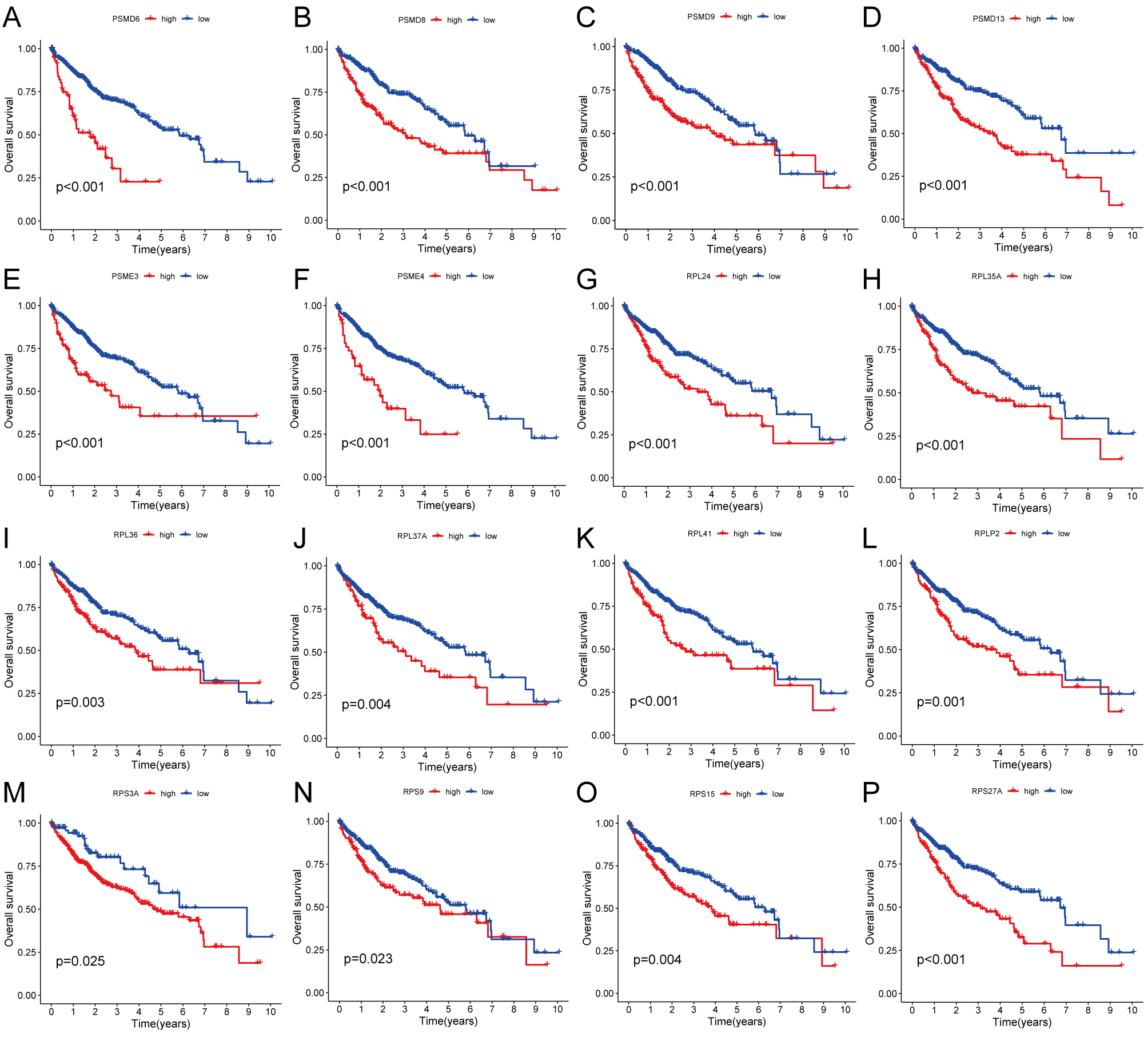

Supplement: Supplementary Figure 2 — (A-P) The Kaplan–Meier curves for survival status and survival time in the high and low expression groups of DRAGs. (A) PSMD6. (B) PSMD8. (C) PSMD9. (D) PSMD13. (E) PSME3. (F) PSME4. (G) RPL24. (H) RPL35A. (I) RPL36. (J) RPL37A. (K) RPL41. (L) RPLP2. (M) RPS3A. (N) RPS9. (O) RPS15. (P) RPS27A. [file Image_2.tif]

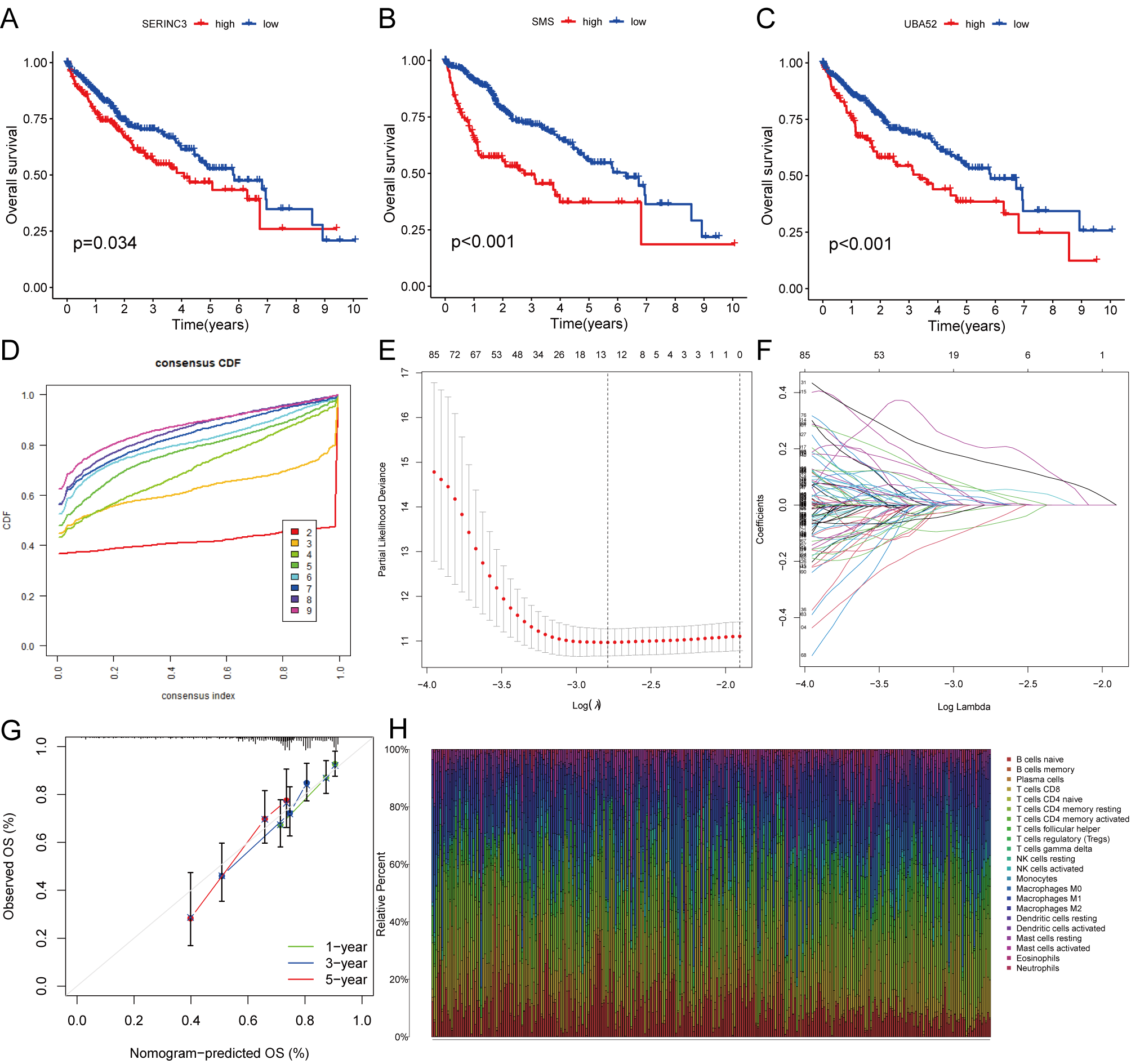

Supplement: Supplementary Figure 3 — (A-E) The Kaplan–Meier curves for survival status and survival time in the high and low expression groups of DRAGs. (A) SERINC3. (B) SMS. (C) UBA52. (D) Cumulative density function (CDF) curves validate the clustering accuracy. (E, F) LASSO regression analysis and consistency test. (G) Calibration plot of the nomogram. (H) Histogram of the percentage of infiltrating immune cells in patients with liver cancer. [file Image_3.tif]
