# Supplementary material for: Typing and modeling of hepatocellular carcinoma based on disulfidptosis-related amino acid metabolism genes for predicting prognosis and guiding individualized treatment
Source: Front Oncol. 2023 Aug 11;13:1204335. doi: 10.3389/fonc.2023.1204335 (PMC10454915; doi:10.3389/fonc.2023.1204335)
Supplement: Supplementary file 4 [file DataSheet_1.zip › Table S7.docx]

Table S7. Statistical results of immunohistochemistry for CD8A protein expression in HCC tissues and normal liver tissues.

|  | **Tissue** | | **χ2**  **P value** |
| --- | --- | --- | --- |
|  | **Normal (n=20)** | **Cancer (n=20)** |  |
| **Immunohistochemical grade** | 12(60%)  8(40%) | 3(15%)  17(85%) | 8.640  0.003 |
| High expression of CD8A  Low expression of CD8A |  |  |  |
